# Preserving Pathways: Strategies for Left Superior Vena Cava Management in Pediatric Cardiac Surgery

**DOI:** 10.1016/j.atssr.2025.03.004

**Published:** 2025-03-19

**Authors:** Chen Chia Wang, Harrison S. Stuart, David P. Bichell, Karla Christian

**Affiliations:** 1 Vanderbilt University Medical Center, Nashville, Tennessee; 2 McGovern Medical School, Houston, Texas

## Abstract

**Background:**

Persistent left superior vena cava (LSVC) is a common congenital anomaly requiring operative management when it causes significant right-to-left shunting or during heart transplantation. Comparative patency of various LSVC repatriation methods to the right side of the heart has not been systematically studied.

**Methods:**

This is a single-institution, retrospective review of patients undergoing surgical management of LSVC from 2013 to 2023. Patients were grouped on the basis of the LSVC to right atrium path: coronary sinus (CS) group with LSVC-CS anastomosis; systemic vein (SV) group with LSVC drainage through donor superior vena cava, right atrial appendage, or innominate vein; or atrial baffle (AB) group. Our primary objective is LSVC patency rate in each group.

**Results:**

Twenty-two patients with a median age of 27 months met inclusion criteria; 4 patients were in the CS group, 9 in the SV group, and 9 in the AB group. At the time of collection, 3 (75%) patients in the CS group, 3 (33%) in the SV group, and 9 (100%) in the AB group showed LSVC patency. All patients demonstrating patency in CS and SV groups were older than 2 years, whereas all patients with occlusion (except for a 35-year-old patient) were younger than 2 years.

**Conclusions:**

Of the methods redirecting LSVC to the right atrium, patency may be best preserved with an undistorted LSVC reunited with a retained CS or redirected by an intracardiac baffle compared with methods that displace the LSVC. Older patients with higher weight may have better patency rates with LSVC reconstruction.

## Visual Abstract

**Figure:**
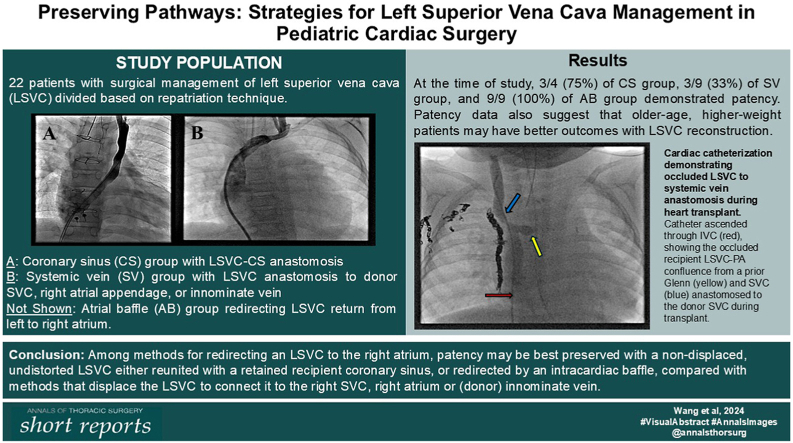

**In Short**

▪ In redirecting a left superior vena cava (LSVC) to the right atrium, patency may be best preserved with a nondisplaced, undistorted LSVC either reunited with a retained recipient coronary sinus or redirected by an intracardiac baffle.
▪ Older age at operation may portend LSVC patency after surgical redirection.

Persistent left superior vena cava (LSVC) occurs in 3% to 10% of individuals with congenital heart disease.^1^ The LSVC drains into the right atrium (RA) through the coronary sinus (CS) in approximately 90% of cases and to the left atrium in 10% of cases.^2^ Surgical redirection of an LSVC to the RA is indicated during significant right-to-left shunting and desaturation, thromboembolic events, or orthotopic heart transplantation (OHT). Several techniques for OHT in patients with LSVC lacking a patent innominate vein have been described, including connection of the LSVC to a donor systemic vein (SV),^3^ intracardiac baffle of LSVC to RA,^4^ and preservation or re-creation of native LSVC-CS drainage by retaining or restoring the native CS pathway within the recipient atrial cuff.^5^ Comparative patency of the various methods of LSVC repatriation to the right side of the heart has not been systematically studied. We hypothesized that LSVC patency is threatened by methods that displace the LSVC and that patency is best preserved with a nondisplaced, undistorted LSVC either reunited with a retained recipient CS or baffled to the RA.

## Patients and Methods

### Patient Population and Data Collection

This study was approved by the Vanderbilt University institutional review board under protocol #150319. This was a retrospective institutional review from April 2013 to October 2023 of patients undergoing surgical management of LSVC. Included were patients with an LSVC presenting for Glenn takedown, creation of an atrial baffle (AB) for unroofed LSVC, or OHT. Excluded were patients with a patent innominate bridging vein. Patency data were up to date as of December 2023 and were analyzed on the basis of postoperative echocardiography, cardiac catheterization, or computed tomography angiography.

### LSVC Management Technique Grouping

The LSVC management techniques were divided into 3 groups: CS group, SV group, and AB group. Surgical techniques that involved drainage of the LSVC into the RA through the native CS were placed in the CS group. Operative managements of LSVC that involved anastomosis to a donor SV were placed in the SV group. Distinguishing characteristics within this group are the SV (superior vena cava [SVC], inferior vena cava, or innominate vein) to which the anastomosis is formed as well as the routing of the anastomosis. All patients in the SV group, therefore, are all OHT patients. Patients managed by creation of an interatrial baffle to redirect venous drainage from the LSVC to the RA were placed in the AB group.

## Results

Twenty-two patients with a median age at operation of 27 months and weight at operation of 11.6 kg met inclusion criteria (Table); 4 (18%) were in the CS group, 9 (41%) were in the SV group, and 9 (41%) were in the AB group. The CS group had a median age of 46 months, weight of 15.3 kg, and bypass time of 281 minutes. The SV group had a median age of 23 months, weight of 9.8 kg, and bypass time of 234 minutes. The AB group had a median age of 27 months, weight of 10.4 kg, and bypass time of 135 minutes. Concomitant procedures during which the LSVC rerouting was performed are shown in the Supplemental Table.

**Table:** Patient Demographics, LSVC Management Technique, and Postoperative LSVC Patency

Patient	Sex	Age (mo)	Weight (kg)	Main Procedure	Study Group	LSVC Drainage	Postoperative Time	Imaging	Patent
1	M	2	5.9	OHT	CS	RA cuff	POM2	CATH	No
2	M	192	78	OHT	SV	SVC	POY6	CATH	Yes
3	F	2	4.1	OHT	AB	RA	POY1	CATH	Yes
4	M	66	17.5	OHT	SV	Innominate vein	POY5	CATH	Yes
5	M	5	7.5	OHT	SV	SVC	POD5	CATH	No
6	F	1	5.0	OHT	SV	Innominate vein	POD24	CATH	No
7	M	4	6.7	OHT	SV	Innominate vein	POD6	CATH	No
8	M	22	8.2	OHT	SV	Innominate vein	POM1	CATH	No
9	F	132	34.9	OHT	CS	CS	POY2	CATH	Yes
10	F	98	25	AB	AB	RA	POD5	TTE	Yes
11	F	59	15.4	AB	AB	RA	POY10	TTE	Yes
12	F	37	13.3	AB	AB	RA	POY8	TTE	Yes
13	M	27	10.4	AB	AB	RA	POD4	TTE	Yes
14	F	5	5.2	AB	AB	RA	POY5	TTE	Yes
15	M	4	3.8	AB	AB	RA	POD30	TTE	Yes
16	M	64	17.9	AB	CS	CS	POM1	CATH	Yes
17	M	241	79	AB	AB	RA	POD1	TTE	Yes
18	F	424	81.9	OHT	SV	IVC	POD5	CATH	No
19	M	23	9.6	OHT	SV	SVC	POM4	CATH	No
20	F	97	22.6	OHT	SV	SVC	POD7	TTE	Yes
21	F	5	4.3	OHT	AB	RA	POM1	CATH	Yes
22	F	27	12.7	Glenn takedown	CS	CS	POM1	CTA	Yes

AB, atrial baffle; CATH, catheterization; CS, coronary sinus; CTA, computed tomography angiography; F, female; IVC, inferior vena cava; LSVC, left superior vena cava; M, male; OHT, orthotopic heart transplantation; POD, postoperative day; POM, postoperative month; POY, postoperative year; RA, right atrium; SV, systemic vein; SVC, superior vena cava; TTE, transthoracic echocardiography.

**Figure 1:**
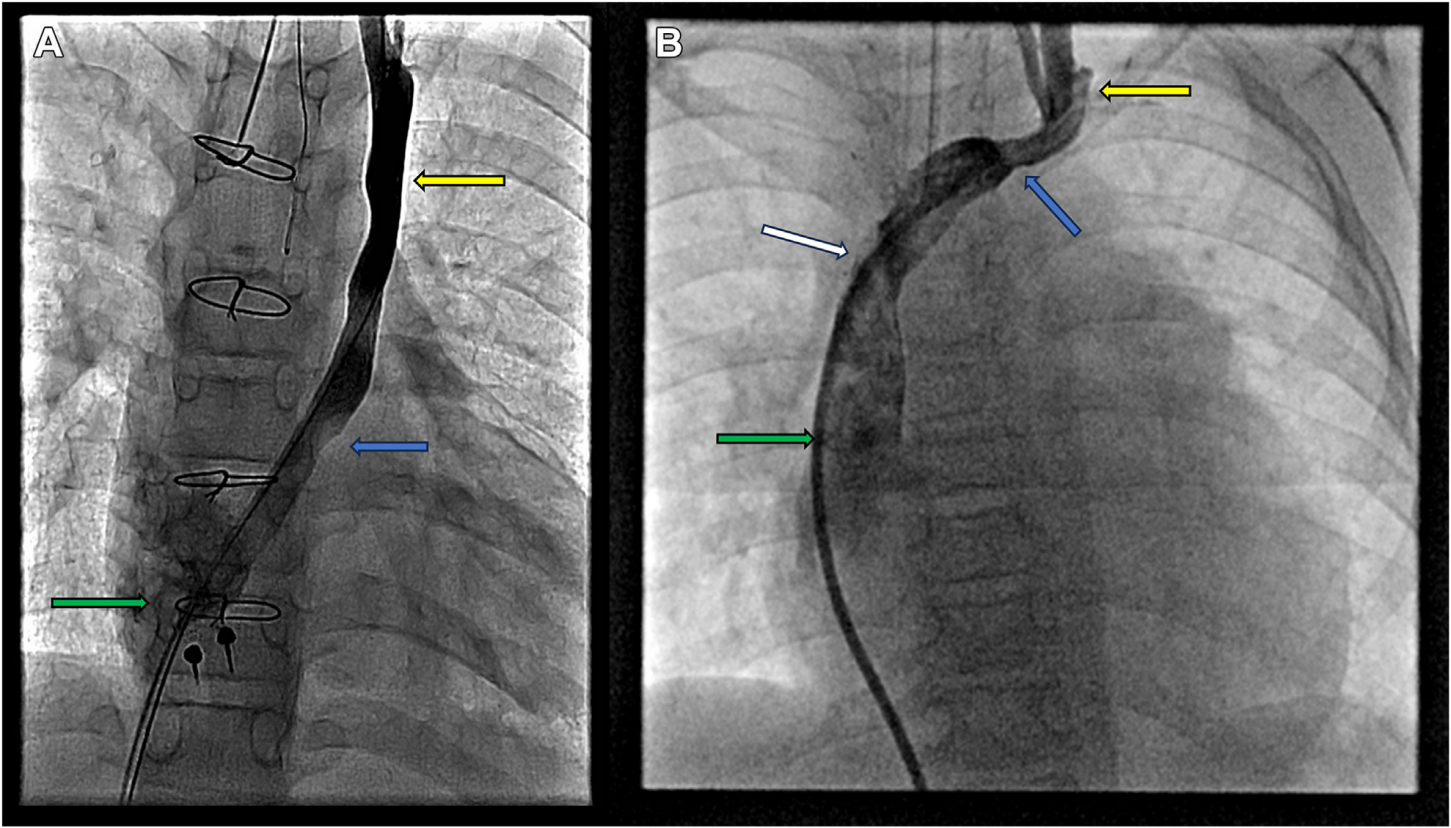
Patent left superior vena cava after heart transplantation. (A) Patent left superior vena cava (yellow arrow) and coronary sinus (blue arrow) anastomosed to donor right atrium (green arrow) in patient 9. (B) Patent left superior vena cava (yellow arrow) anastomosed to the donor innominate vein (blue arrow), which drains into the donor superior vena cava (white arrow) and right atrium (green arrow) in patient 4.

**Figure 2:**
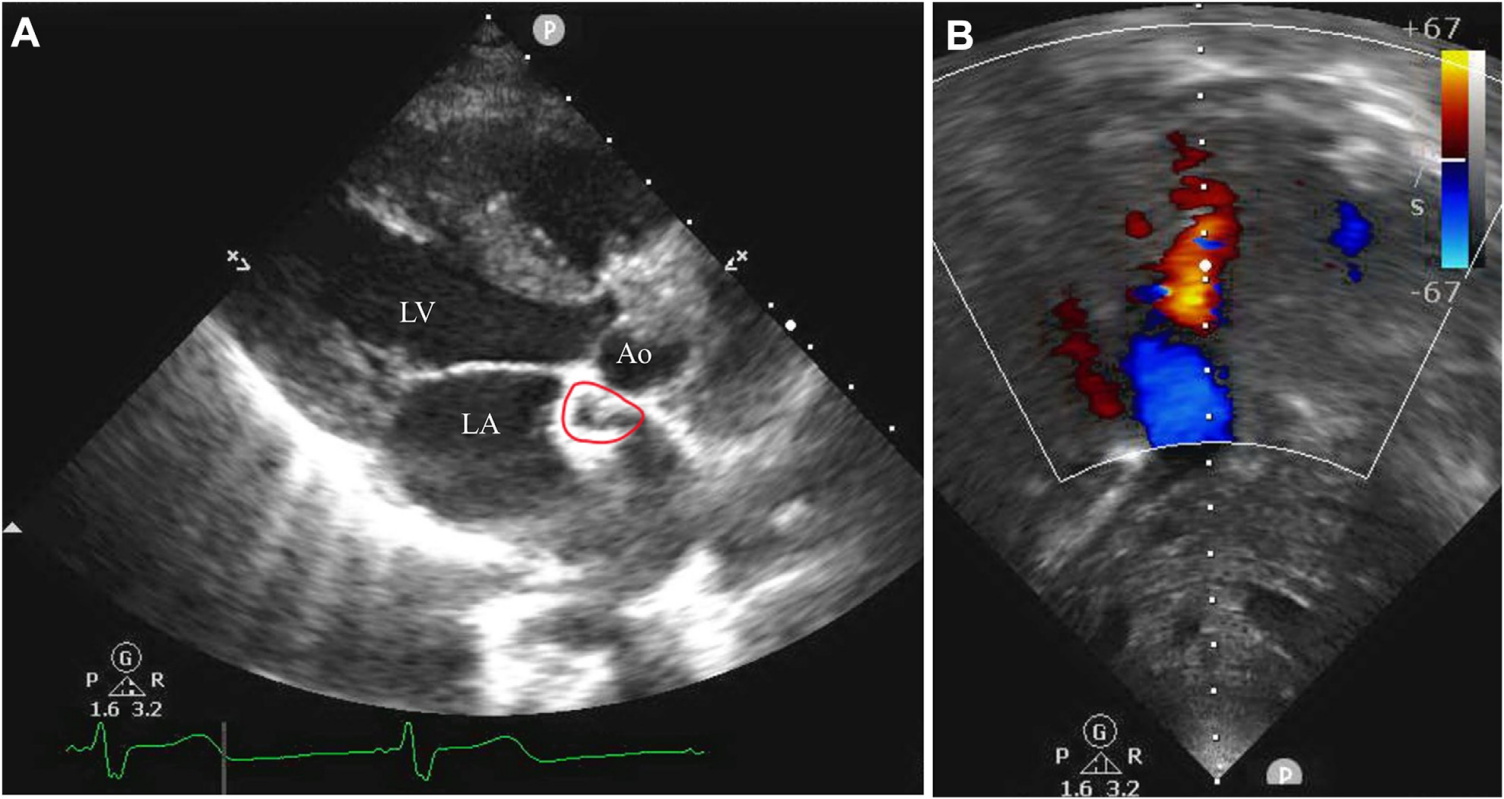
Echocardiogram demonstrating patency of left superior vena cava–right atrial baffle in patient 12. (A) Parasternal long-axis view demonstrating atrial baffle (red circle) near the superior aspect of the left atrium. (B) Doppler ultrasound demonstrating flow through the patent atrial baffle. (Ao, aorta; LA, left atrium; LV, left ventricle.)

**Figure 3:**
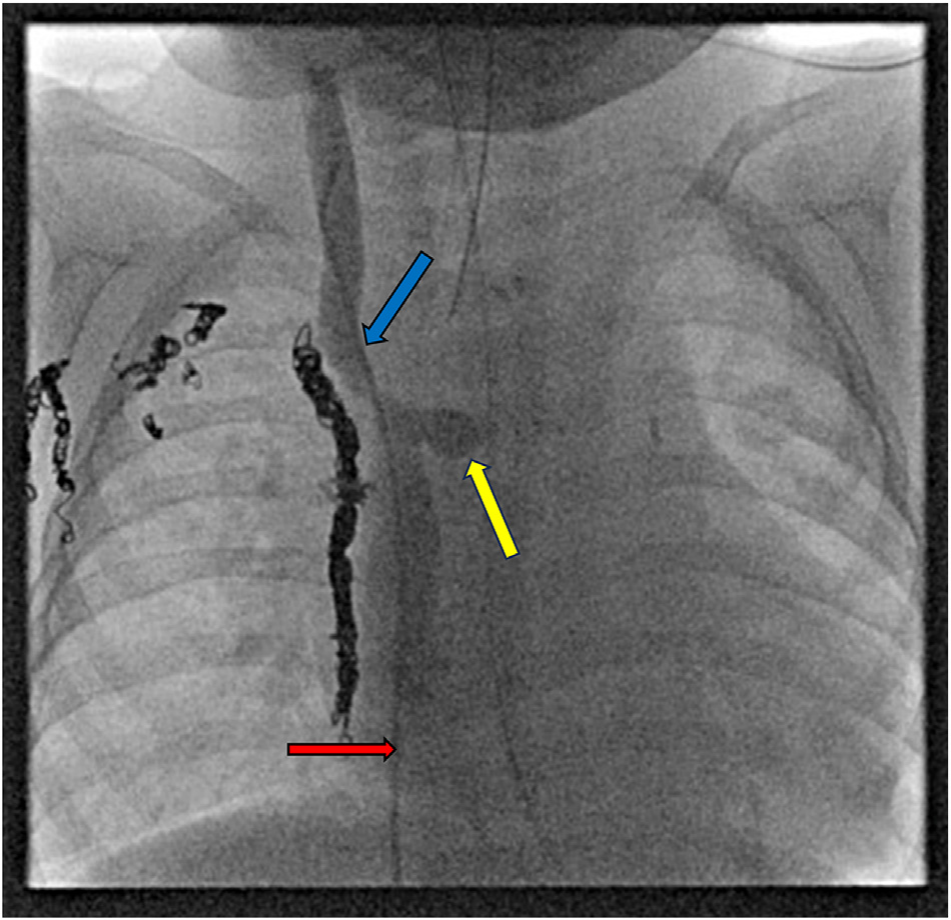
Cardiac catheterization demonstrating occluded anastomosis of left superior vena cava (LSVC) to systemic vein in patient 19 after heart transplantation. Catheter ascended through inferior vena cava (red arrow), showing the occluded recipient LSVC–pulmonary artery confluence (yellow arrow) from a prior bidirectional Glenn and recipient superior vena cava (blue arrow) anastomosed to the donor superior vena cava during transplantation. Occlusion is demonstrated by lack of retrograde flow of contrast material to the LSVC.

During the time of data collection, 3 (75%) patients in the CS group showed LSVC patency, 3 (33%) patients in the SV group showed patency, and 9 (100%) patients in the AB group showed patency. Of our 22 patients, 4 (17%) patients were deceased, with 2 (22%) in the SV group and 2 (22%) in the AB group. Representative images of patent connections are shown in Figures 1 and 2, and a representative image of an occluded connection is shown in Figure 3.

## Comment

This case series serves to elucidate certain trends in outcomes that may help guide future LSVC management and provides a basis for broader future study. Analysis of postoperative imaging studies to determine continued LSVC patency revealed differential but mixed patency patterns between the groups. The clinical impact of LSVC patency needs to be further elucidated to determine the utility of optimizing patency rates during operative LSVC management and optimizing postoperative LSVC patency surveillance.

All patients in the AB group demonstrated patent LSVC at their most recent imaging study. Another single-center study evaluating the management of unroofed CS syndrome with LSVC using an AB found similarly excellent patency rates.^6^ In the CS group, 3 of 4 patients demonstrated LSVC patency as many as 2 years postoperatively. The native LSVC-CS vasculature of the 1 patient (patient 1) who demonstrated occlusion was left in situ. Initial echocardiography demonstrated patency of the reconstruction on postoperative day 12, with subsequent catheterization demonstrating occlusion at 2 months. Notably, patient 1 was 2 months old at the time of operation, whereas the 3 patients who demonstrated patency (patients 9, 16, and 22) were around 10, 2, and 2 years of age at the time of operation. In the SV group, 3 of 9 patients demonstrated LSVC patency as far as 6 years after their operation. Of the 6 patients with occluded vessels, the novel LSVC structures were found to be occluded as soon as 5 days (patient 5) and as late as 4 months (patient 19) postoperatively. The LSVC-SV techniques demonstrating patency include an end-to-end LSVC-SVC anastomosis with side-to-side right SVC (RSVC)–SVC connection (patient 2), an end-to-end ante-aortic LSVC-innominate anastomosis with end-to-end RSVC-SVC anastomosis (patient 4), and an LSVC-RSVC anastomosis by the native pulmonary artery confluence (patient 20). Of note, the 3 patients demonstrating patency were all older than 5 years at the time of operation. All SV group patients with occlusion, except patient 18 (who was older than 35 years), were younger than 2 years at operation.

Multiple factors are likely to influence the occlusion of novel connections between a donor SV and an LSVC after OHT. Rerouting of the LSVC introduces the risk of distortion of the vein, and mobilization across the aorta involves some degree of narrowing from stretching. This risk of stenosis is compounded by compression of the high-pressure aorta on the low-pressure venous structure. Older patients with higher weight may also have better outcomes with LSVC reconstruction.

An interesting point of discussion is the clinical importance of LSVC patency. In patients with bilateral SVCs undergoing OHT, simply ligating the LSVC instead of reconstructing an anastomosis is a viable option when the risk of cerebral venous congestion is minimal.^7^ However, LSVC occlusion can disrupt physiologic venous drainage in cases in which the patient does not have an RSVC. LSVC patency is also crucial in cases involving a risk of cerebral venous congestion^7^ or retrograde flow draining the CS to the innominate vein.^8^ It is also possible that the patient’s age could affect the clinical impact of LSVC nonpatency as venous collaterals may develop in younger patients in the setting of LSVC occlusions. In our case series, only 2 of 4 deaths had a nonpatent LSVC; the causes of both were unrelated to LSVC occlusion. Furthermore, the surviving patients with LSVC occlusion did not have significant clinical events as a result of the occlusion. Therefore, the clinical significance of LSVC patency in diverse patient anatomies remains an interesting area of investigation.

Another important aspect of LSVC management is the postoperative follow-up. Cardiac catheterization and computed tomography produce accurate imaging of restructured LSVC pathways, although inconsistently visualized unless specifically sought. Routine posttransplantation echocardiography may be insufficient to document the patency of reconstructed LSVCs, and attention is still needed on the provider’s end to specifically look for the LSVC connections on echocardiography, cardiac catheterization, or computed tomography angiography.

Data trends in this small, heterogeneous population suggest that the best operative strategy to ensure prolonged patency of an LSVC in OHT patients may be to preserve or to restore an undistorted LSVC connection to a native CS. Alternative approaches that create an anastomosis between the LSVC and donor SV appear more prone to LSVC occlusion. Only in 3 patients, all older than 5 years at operation, did this technique for reconstruction provide durable venous drainage of the LSVC. For correction of an unroofed LSVC draining into the left atrium, an interatrial baffle can successfully redirect venous flow back to the RA with acceptable long-term patency.

### Limitations

As a case series, our study lacks statistical power for us to determine any significant effects of each technique and to rule out confounding variables, such as patient age or weight at surgery. Because the time of operations ranges from 2013 to 2023 and we looked at each patient’s most recent reports, there is variability in the time frames in which occlusion may take place and be observed. Because this is a heterogeneous cohort of patients managed by various surgeons, there was also a lack of standardization with surgical techniques used. Future studies with larger sample sizes would, it is hoped, elucidate any differences between LSVC management techniques and provide insight on the clinical significance of LSVC patency rates.

### Conclusion

Of the methods for redirecting an LSVC to the RA, patency may be best preserved with a nondisplaced, undistorted LSVC either reunited with a retained recipient CS or redirected by an intracardiac baffle compared with methods that displace the LSVC to connect it to RSVC, RA, or (donor) innominate vein.
