# Supplementary material for: Preserving Pathways: Strategies for Left Superior Vena Cava Management in Pediatric Cardiac Surgery
Source: Ann Thorac Surg Short Rep. 2025 Mar 19;3(3):651–6. doi: 10.1016/j.atssr.2025.03.004 (PMC12559576; doi:10.1016/j.atssr.2025.03.004)
Supplement: Supplementary Table 1 [file mmc1.docx]

**Supplemental Table 1: Additional Patient Operative Characteristics**

| Patient | Study Group | Main Procedure | Bypass (mins) | LSVC Drainage | Additional Procedures |
| --- | --- | --- | --- | --- | --- |
| 1 | CS | OHT | 152 | RA cuff | Aortic arch repair |
| 2 | SV | OHT | 229 | SVC | None |
| 3 | AB | OHT | 330 | RA | Aortic arch augmentation, VAD explant, PA band removal |
| 4 | SV | OHT | 230 | Innominate vein | PA reconstruction |
| 5 | SV | OHT | 214 | SVC | PA reconstruction, ECMO decannulation |
| 6 | SV | OHT | 234 | Innominate vein | ECMO re-cannulation |
| 7 | SV | OHT | 329 | Innominate vein | PA reconstruction, PAPVC repair, ECMO cannulation for ventricular dysfunction |
| 8 | SV | OHT | 361 | Innominate vein | Aortic arch reconstruction, PA reconstruction, penny removal from esophagus |
| 9 | CS | OHT | 230 | CS | PA reconstruction |
| 10 | AB | Atrial baffle | 100 | RA | ASD repair, secundum, primary surgical closure |
| 11 | AB | Atrial baffle | 85 | RA | ASD repair, secundum |
| 12 | AB | Atrial baffle | 155 | RA | ASD closure, MPA patch for dilation, resection of pulm vein confluence |
| 13 | AB | Atrial baffle | 135 | RA | ASD repair, AVC (AVSD) repair, with cleft MV repair, complete closure of "cleft" |
| 14 | AB | Atrial baffle | 119 | RA | ASD repair, secundum, patch, VSD repair, line placement |
| 15 | AB | Atrial baffle | 410 | RA | VSD repair, PA debanding, line placement (transthoracic, right atrium), tricuspid valvuloplasty, |
| 16 | CS | Atrial baffle | 344 | CS | VSD enlargement, DORV repair, PA reconstruction |
| 17 | AB | Atrial baffle | 74 | RA | None |
| 18 | SV | OHT | 304 | IVC | PA reconstruction |
| 19 | SV | OHT | 359 | SVC | None |
| 20 | SV | OHT | 169 | SVC | None |
| 21 | AB | OHT | 232 | RA | Atrial level switch, PA reconstruction, takedown aortopulmonary shunt, double lumen transthoracic line placement |
| 22 | CS | Glenn Takedown | 331 | CS | AVSD repair, tricuspid and mitral valvuloplasty, closure of CS ASD, partial closure of secundum ASD, DCRV repair |
